# Characterization of D-Arabitol as Newly Discovered Carbon Source of *Bacillus methanolicus*

**DOI:** 10.3389/fmicb.2019.01725

**Published:** 2019-07-31

**Authors:** Marina Gil López, Marta Irla, Luciana F. Brito, Volker F. Wendisch

**Affiliations:** ^1^Genetics of Prokaryotes, Faculty of Biology and CeBiTec, Bielefeld University, Bielefeld, Germany; ^2^Department of Biotechnology and Food Science, Norwegian University of Science and Technology, Trondheim, Norway

**Keywords:** *Bacillus methanolicus*, differential transcriptome analysis, mannitol metabolism, arabitol metabolism, monophasic growth, operon organization

## Abstract

*Bacillus methanolicus* is a Gram-positive, thermophilic, methanol-utilizing bacterium. As a facultative methylotroph, *B. methanolicus* is also known to utilize D-mannitol, D-glucose and, as recently discovered, sugar alcohol D-arabitol. While metabolic pathways for utilization of methanol, mannitol and glucose are known, catabolism of arabitol has not yet been characterized in *B. methanolicus*. In this work we present the elucidation of this hitherto uncharted pathway. In order to confirm our predictions regarding genes coding for arabitol utilization, we performed differential gene expression analysis of *B. methanolicus* MGA3 cells grown on arabitol as compared to mannitol via transcriptome sequencing (RNA-seq). We identified a gene cluster comprising eight genes that was up-regulated during growth with arabitol as a sole carbon source. The RNA-seq results were subsequently confirmed via qRT-PCR experiments. The transcriptional organization of the gene cluster identified via RNA-seq was analyzed and it was shown that the arabitol utilization genes are co-transcribed in an operon that spans from BMMGA3_RS07325 to BMMGA3_RS07365. Since gene deletion studies are currently not possible in *B. methanolicus*, two complementation experiments were performed in an arabitol negative *Corynebacterium glutamicum* strain using the four genes discovered via RNA-seq analysis as coding for a putative PTS for arabitol uptake (BMMGA3_RS07330, BMMGA3_RS07335, and BMMGA3_RS07340 renamed to *atlABC*) and a putative arabitol phosphate dehydrogenase (BMMGA3_RS07345 renamed to *atlD*). *C. glutamicum* is a natural D-arabitol utilizer that requires arabitol dehydrogenase MtlD for arabitol catabolism. The *C. glutamicum mtlD* deletion mutant was chosen for complementation experiments. Heterologous expression of *atlABCD* as well as the arabitol phosphate dehydrogenase gene *atlD* from *B. methanolicus* alone restored growth of the *C. glutamicum* Δ*mtlD* mutant with arabitol. Furthermore, D-arabitol phosphate dehydrogenase activities could be detected in crude extracts of *B. methanolicus* and these were higher in arabitol-grown cells than in methanol- or mannitol-grown cells. Thus, *B. methanolicus* possesses an arabitol inducible operon encoding, amongst others, a putative PTS system and an arabitol phosphate dehydrogenase for uptake and activation of arabitol as growth substrate.

## Introduction

*Bacillus methanolicus* is an aerobic, Gram-positive, thermophilic, methanol-utilizing bacterium originally isolated from freshwater marsh soil (Schendel et al., 1990; Arfman et al., 1992). Methylotrophs, such as *B. methanolicus*, utilize carbon sources without C-C bonds also called C1 substrates. The key intermediate for biological C1 fixation is formaldehyde, and *B. methanolicus* belongs to the group of facultative methylotrophs that fix formaldehyde via the ribulose monophosphate (RuMP) cycle (Anthony, 1982; Arfman et al., 1992). What makes methanol an attractive feedstock is the fact that it is abundant and cheap, and that addition of methanol to fermentation broth reduces the risk of microbial contamination in fermentative processes due to toxicity of its derivative – formaldehyde (Irla et al., 2015; Müller et al., 2015a). Furthermore, methanol presents a non-food alternative to conventional feedstock generally used in biotechnological processes. The ability to utilize methanol as carbon source, in addition to its high growth temperature, makes *B. methanolicus* MGA3 a promising candidate for biotechnological amino acid production. It has been successfully used for methanol-based production of the amino acids L-lysine and L-glutamate (Brautaset et al., 2007), and has been engineered for production of the compounds cadaverine (Naerdal et al., 2015; Irla et al., 2016) and γ-aminobutyric acid (GABA) (Irla et al., 2017). Furthermore, tools for gene expression have been recently developed, comprising gene co-expression from two different plasmids and controlled inducible gene expression systems with both rolling circle and theta replicating plasmids (Irla et al., 2016).

**FIGURE 1:**
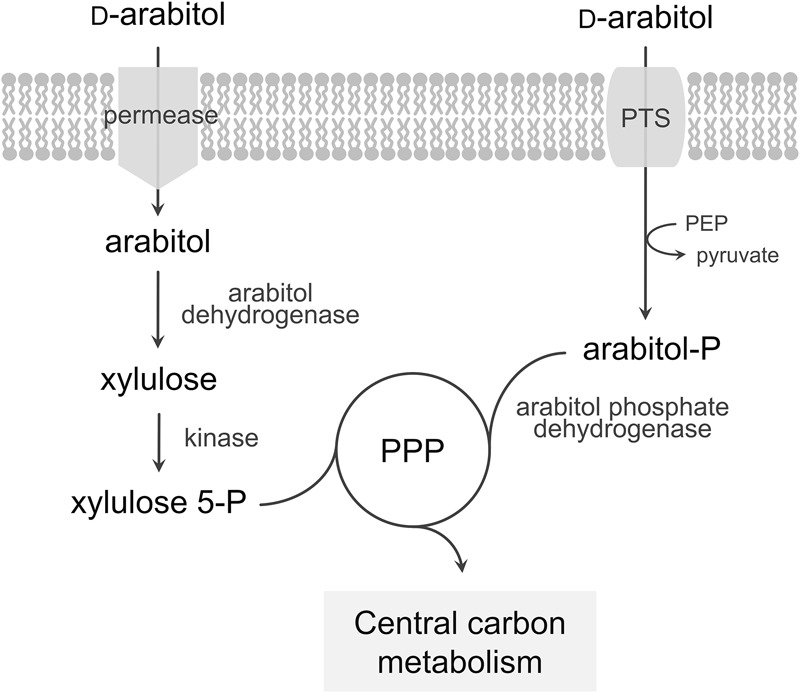
Model of the pathways for arabitol catabolism in bacteria. The first route represents uptake of arabitol via a permease, a dehydrogenase and a kinase while the second route represents uptake via a PTS and a phosphate dehydrogenase. PTS, phosphotransferase system; PEP, phosphoenolpyruvate; PPP, pentose phosphate pathway.

*Bacillus methanolicus* methylotrophy has been extensively studied and characterized in recent years. This contributed to a broader understanding of methanol metabolism and its regulation by fully sequencing the MGA3 genome (Heggeset et al., 2012; Irla et al., 2014), achieving a comprehensive analysis of the transcriptional landscape using RNA-seq (Irla et al., 2015) and accomplishing proteome (Müller et al., 2014) and metabolome (Müller et al., 2015b; Carnicer et al., 2016) studies. These studies not only increased our understanding of methanol metabolism in *B. methanolicus*, but also yielded insight into catabolic pathways of alternative carbon sources. *B. methanolicus* is known to utilize D-mannitol and D-glucose as sole carbon and energy sources and metabolic pathways for the utilization of these substrates have already been described (Heggeset et al., 2012). Both mannitol and glucose enter the cells via a phosphotransferase system (PTS) as mannitol 1-phosphate and glucose 6-phosphate, respectively, and are converted to fructose 6-phosphate. Here, we describe and characterize utilization of the pentose sugar alcohol D-arabitol for growth of *B. methanolicus* MGA3. Two alternative pathways for arabitol utilization have been described in bacteria (Figure 1): uptake of arabitol via a permease followed by intracellular oxidation and phosphorylation to yield xylulose 5-phosphate, route described for proteobacteria and actinobacteria as in e.g., *Corynebacterium glutamicum* (Laslo et al., 2012), *Enterobacter aerogenes* (Charnetzky and Mortlock, 1974), *Klebsiella pneumoniae* (Heuel et al., 1997), *Rhizobium trifolii* (Primrose and Ronson, 1980), and *Pseudomonas fluorescens* (Brünker et al., 1998), or PTS-mediated uptake and phosphorylation followed by oxidation to pentose phosphates as described for the firmicutes *Listeria moncytogenes* (Kentache et al., 2016) and *Enterococcus avium* (Povelainen et al., 2003).

Arabitol is ubiquitous in nature and has been found in plants and fungi, often alongside mannitol, being involved in osmoprotection and carbohydrate storage (Daly et al., 1967; Plemenitaš et al., 2008; Wang et al., 2019). It has additionally been reported that arabitol conferred drought tolerance when provided by a lichenous fungi to the green algae *Trebouxia* sp. (Kosugi et al., 2013). Yeast or yeast-like fungi produce extracellular glycolipids, some of which have been reported to exceptionally contain mannitol and arabitol residues (Kulakovskaya and Kulakovskaya, 2014b). Their role in metabolism includes promotion of solubilization and absorption of hydrophobic substrates, extracellular reserve of carbon sources and antibiotic activity (Kulakovskaya and Kulakovskaya, 2014a). Roselipins, consisting of C_20_-fatty acids with three hydroxyl groups, mannose and arabitol residues, are extracellular glycolipids synthesized by *Clonostachys rosea* (Tabata et al., 1999). Mannosylerythritol lipids are major extracellular glycolipids of the *Pseudozyma* genera. In *Pseudozyma parantarctica* the rarely occurring mannosylarabitol lipids and mannosylmannitol lipids have been described (Morita et al., 2009, 2012). The natural ecological niches of these extracellular glycolipid yeast producers include soil, nectaries and leaves of plants (Kulakovskaya and Kulakovskaya, 2014a), coexisting together with *B. methanolicus* in similar habitats. Additionally, some yeasts possess the ability of transforming glucose into arabitol (Kordowska-Wiater, 2015). It has previously been reported that methanol is a by-product of pectin metabolism during cell wall synthesis and pathogen attack in plants, which in turn assists in plant immunity (Fall and Benson, 1996; Komarova et al., 2014). Bacterial isolates from leaf surfaces showed the presence of *B. methanolicus* on *Citrus paradisi* plants (Izhaki et al., 2013). These findings indicate that methanol, mannitol, glucose and arabitol might be present in the natural habitat of *B. methanolicus*.

In the present study, we characterized growth of *B. methanolicus* MGA3 on arabitol. Based on our differential transcriptome analysis, the genes coding for proteins involved in arabitol utilization were identified, and their functionality confirmed by genetic complementation and enzyme assays.

## Materials and Methods

### Bacterial Strains, Media and Cultivation Conditions

The strains used in this study are listed in Table 1. *C. glutamicum* ATCC 13032 was used as the expression host and *Escherichia coli* DH5α was used as the general cloning host. *E. coli* strains were routinely cultivated at 37°C and 180 rpm in Lysogeny Broth (LB) media or on LB plates [1% (w/v) agar] supplemented with 25 μg mL^-1^ kanamycin if relevant. *B. methanolicus* strains were cultivated at 50°C and 200 rpm in minimal MVcMY media for pre-cultures or MVcM for main cultures as previously described (Brautaset et al., 2003) with 200 mM methanol, 5, 10, 15, and 50 mM mannitol or 5, 10, 15, 30, and 60 mM arabitol. For co-consumption experiments, 15 mM mannitol and 15 mM arabitol were added to the media. Main cultures of all *B. methanolicus* experiments were inoculated at a start optical density (OD_600_) of 0.2. *C. glutamicum* strains were routinely cultivated at 30°C and 120 rpm in LB media with 30 mM glucose for pre-cultures and in minimal CGXII media (Eggeling and Bott, 2005) for main cultures with 30 mM glucose or 30 mM arabitol. Media were supplemented with 25 μg mL^-1^ kanamycin when necessary and 1 mM IPTG was added for induction of gene expression at inoculation of the main cultures, which was done at an initial OD_600_ of 0.5. Cultivations were performed in 500 mL baffled shake flasks with 50 mL media volume and in biological triplicates in all cases.

**Table 1:** Strains and plasmids used in this study.

Strain or plasmid	Relevant characteristics	References
**Strains**		
*E. coli* DH5α	F^-^ *thi*-1 *endA1 hsdR17*(r^-^, m^-^) *supE44* Δ*lacU169* (φ80*lacZ*ΔM15) *recA1 gyrA96 relA1*	Hanahan, 1983
*C. glutamicum* ATCC 13032	Wild type strain	Abe et al., 1967
*C. glutamicum* RES167 Δ*mtlD*	*mtlD* deletion mutant of *C. glutamicum* RES167	Laslo et al., 2012
*B. methanolicus* MGA3	Wild type strain (ATCC 53907)	Schendel et al., 1990
*B. methanolicus* PB1	Wild type strain (ATCC 51375, NCIMB13113)	Arfman et al., 1992
**Plasmids**		
pVWEx1	Km^R^; *E. coli*/*C. glutamicum* shuttle vector for regulated gene expression (P_tac_, *lacI*^q^, pCG1 *oriV_Cg_*)	Peters-Wendisch et al., 2001
pVWEx1-*atlABCD*	pVWEx1 derivative for IPTG-inducible expression of BMMGA3_RS07330, BMMGA3_RS07335, BMMGA3_RS07340 and BMMGA3_RS07345 (*atlABCD*) from *B. methanolicus* MGA3	This study
pVWEx1-*atlD*	pVWEx1 derivative for IPTG-inducible expression of BMMGA3_RS07345 (*atlD*) from *B. methanolicus* MGA3	This study
pVWEx1-*atlABCDEF*	pVWEx1 derivative for IPTG-inducible expression of BMMGA3_RS07330, BMMGA3_RS07335, BMMGA3_RS07340, BMMGA3_RS07345, BMMGA3_RS07350, and BMMGA3_RS07355 (*atlABCDEF*) from *B. methanolicus* MGA3	This study

*Km^*R*^, kanamycin resistance.*

### Recombinant DNA Work

The description of all plasmids constructed in this study is presented in Table 1. Molecular cloning was performed as described by Sambrook and Russell (2001). Primer sequences used in this study were obtained from Metabion (Planegg/Steinkirchen, Germany) and are listed in Supplementary Table 1. Total DNA isolation from *B. methanolicus* MGA3 was performed as previously described (Eikmanns et al., 1994). Inserts were amplified by polymerase chain reactions (PCRs) using ALLin^TM^ HiFi DNA Polymerase (HighQu, Kraichtal, Germany) and purified with the NucleoSpin^®^ Gel and PCR Clean-up kit (Macherey-Nagel, Düren, Germany). For plasmid isolation, the GeneJET Plasmid Miniprep Kit (Thermo Fisher Scientific, Waltham, MA, United States) was used. Ends of DNA fragments (PCR-amplified fragments and plasmid pVWEx1 cut with restriction enzymes) were joined by means of the isothermal DNA assembly method (Gibson et al., 2009). Transformation of chemically competent *E. coli* cells was done following the procedure of Mandel and Higa (1970). Colony PCRs were performed using the Taq polymerase (New England Biolabs, Ipswich, England) with primers acF1, acR1 and acR2 (Supplementary Table 1). All cloned DNA fragments were verified by sequencing (Sequencing Core Facility, Bielefeld University). *C. glutamicum* competent cells and electroporation were prepared as previously described (Eggeling and Bott, 2005).

### Heterologous Expression of *B. methanolicus* Genes in *C. glutamicum*

Plasmids for inducible gene expression in *C. glutamicum* were constructed on the basis of pVWEx1 (Peters-Wendisch et al., 2001). The *atlABCD* and *atlABCDEF* genes were PCR-amplified from *B. methanolicus* MGA3 genomic DNA using the primers acF1 and acR1 or acF1 and acR2, respectively, and the *atlD* gene was amplified using primers aPDF and aPDR (Supplementary Table 1). The resulting PCR product was joined with *Bam*HI digested pVWEx1 via Gibson assembly.

### Isolation of Total RNA

In order to perform total RNA extraction from *B. methanolicus* MGA3 cells, the NucleoSpin RNA isolation Kit (Machery-Nagel, Düren, Germany) and the RNase-free DNase set (Qiagen, Hilden, Germany) were used according to the manufacturer’s instructions. *B. methanolicus* cultures were grown in minimal MVcM media containing 200 mM methanol, 15 mM mannitol or 15 mM arabitol. Cells were harvested in the middle of the exponential growth phase at an OD_600_ of 1.0 followed by total RNA isolation individually for each cultivation condition. The RNA material was tested for contaminating DNA using primers PRIF and PRIR for the amplification of the *proI* gene and primers MRF1 and MRR1 for the amplification of the *mtlR* gene (Supplementary Table 1). No product was obtained for any of the tested samples (data not shown). The quality of the samples was subsequently verified by capillary gel electrophoresis (Agilent Bioanalyzer 2100 system using the Agilent RNA 6000 Pico kit; Agilent Technologies, Böblingen, Germany) and the concentration checked by DropSense^TM^ 16 (Trinean, Ghent, Belgium). The RNA material was subsequently used either for RNA-seq analysis, qRT-PCR or RT-PCR analysis of operon structure.

### cDNA Library Preparation, RNA-Seq and Mapping of Generated RNA-Seq Data

Isolated RNA samples from *B. methanolicus* MGA3 were pooled in equal parts and the total RNA was subsequently used for the cDNA library preparation. The library was prepared and sequenced on a single flow cell of a MiSeq Desktop Sequencer system (Illumina, San Diego, CA, United States) in paired-end mode following a protocol that allowed for the analysis of the whole transcriptome (Mentz et al., 2013). Previous to mapping of the generated reads onto the reference genome, the sequences were trimmed using the tool Trimmomatic version 0.33 (Bolger et al., 2014) to a minimal length of 35 base pairs. The trimmed reads were mapped to the *B. methanolicus* MGA3 reference sequences of the chromosome as well as the two plasmids pBM19 and pBM69 (GenBank accession numbers CP007739, CP007741, and CP007740, respectively) using the software for short read alignment Bowtie (Langmead et al., 2009). For the visualization of the mapped reads the ReadXplorer software was used (Hilker et al., 2014). The differential gene expression analysis was performed with the statistical method DESeq (Anders and Huber, 2010) using the same software. In order to designate a gene as differentially expressed, the cut-off values were set to a change in expression level higher than 30, for which the *P*-value was adjusted to be equal to or less than 0.01. Sequences of differentially expressed genes that coded for proteins of unknown function were subjected to BLASTx analysis for identification of protein family conservations (Altschul et al., 1990).

### Real-Time Quantitative Reverse Transcription PCR (qRT-PCR)

qRT-PCR was performed in order to validate the generated data in RNA-seq analysis. Isolated RNA samples from *B. methanolicus* MGA3 grown on either mannitol, arabitol or a combination of both were used as template. The optimization of a qRT-PCR protocol included a series of standard PCRs using different primer concentrations (250, 400, and 600 mM) and different annealing temperatures (48 to 65°C). For each gene to be analyzed, a pair of primers were designed for the amplification of about 200 bp using the primer design tool of Clone Manager 9 (Scientific & Educational Software, Denver, CO, United States) (Supplementary Table 1). The experiments were performed with the LightCycler^®^ 96 System (Roche Diagnostics, Penzberg, Germany) using the SensiFAST^TM^ SYBR^®^ No-ROX One-Step Kit (Bioline, Luckenwalde, Germany) according to the manufacturer’s instructions. After the optimization process, each reaction mixture contained 400 mM of each primer and 50 ng of RNA in a final volume of 20 μL. The qRT-PCR profile was chosen to be performed as follows: the reverse transcription was performed at 45°C for 10 min, the polymerase activation at 95°C for 2 min followed by 40 cycles of a three-step amplification composed of a denaturation step at 95°C for 5 s, annealing at 55°C for 10 s and extension at 72°C for 5 s and, lastly, dissociation curve analyses were done from 65°C up to 95°C in 0.5°C increments for 5 s each step. Amplification of *repB*, the pBM19 replication initiator gene, was used for sample normalization following the recommendations of Jakobsen et al. (2006). They could confirm by qRT-PCR that the *repB* transcript levels were similar in cells utilizing mannitol and methanol and, additionally, our RNA-seq data showed similar *repB* expression between mannitol and arabitol, too (data not shown). Relative quantification was done by means of the comparative threshold cycle method: the 2^-ΔΔ^*^CT^* method (Livak and Schmittgen, 2001). All measurements were performed in technical replicates.

### Reverse Transcription PCR (RT-PCR) Analysis of Operon Structure

Analysis of the transcriptional organization of the arabitol gene cluster was done via RT-PCR. Isolated RNA from *B. methanolicus* MGA3 grown on arabitol was used as template for cDNA synthesis using the BioScript^TM^ Reverse Transcriptase kit (Bioline, Luckenwalde, Germany) according to the manufacturer’s instructions with gene specific primer RT03, which hybridizes in BMMGA3_RS07365 (Supplementary Table 1). In order to detect if the referred gene cluster was transcribed as a single mRNA molecule, the resulting cDNA was then used as template for PCR using primers spanning gene borders of all genes putatively present in the arabitol gene cluster (Supplementary Table 1).

### Arabitol Phosphate Dehydrogenase Enzymatic Assay of *B. methanolicus* Crude Extracts

*Bacillus methanolicus* MGA3 cells were grown in minimal media with methanol, mannitol or arabitol and collected by centrifugation at 4°C and 4,000 rpm for 10 min in the middle of the exponential growth phase at an OD_600_ of around 1.0. The pelleted cells were re-suspended in Tris-HCl buffer (pH 7.2) and disrupted by sonication (UP 200 S, Dr. Hielscher GmbH, Teltow, Germany) on ice at an amplitude of 55% and a duty cycle of 0.5 for 9 min with a 30 s pause in between. To obtain lysates, the samples were centrifuged for 60–90 min at 4°C and 14,000 rpm to remove cell debris, the supernatants were then collected and protein concentration was determined by means of the Bradford method (Bradford, 1976) using bovine serum albumin as reference. Determination of the arabitol phosphate dehydrogenase activity in the reductive reaction using xylulose 5-phosphate as substrate was performed following the indications of Povelainen et al. (2003). The assay mixture contained 20 mM Tris-HCl buffer (pH 7.2), 1 mM DTT, 0.3 mM NADH, 50–200 μL of crude extract and 0.2–2 mM xylulose 5-phosphate in a total volume of 1 mL. The oxidation rate of NADH was monitored at 340 nm and 30°C for 3 min on a Shimadzu UV-1202 spectrophotometer (Shimadzu, Duisburg, Germany).

### Analysis of Culture Supernatants by Liquid Chromatography

For the quantification of growth substrates mannitol and arabitol in a cultivation broth a high-pressure liquid chromatography (HPLC) system was used (1200 series, Agilent Technologies Deutschland GmbH, Böblingen, Germany) as in Pérez-García et al. (2016). The supernatants of the cell cultures were obtained by centrifugation at 14,000 rpm and at room temperature for 15 min. The analysis was carried out using a column for organic acids (300 mm × 8 mm, 10 μm particle size, 25 Å pore diameter, CS Chromatographie Service GmbH, Langerwehe, Germany) with mobile phase of 5 mM sulphuric acid at a flow rate of 0.8 mL min^-1^, at 60°C and for 17 min. The detection was executed with a refractive index detector (RID G1362A, 1200 series, Agilent Technologies).

## Results

### Growth of *B. methanolicus* MGA3 on Arabitol as Single Carbon Source and as Co-substrate to Mannitol

**FIGURE 2:**
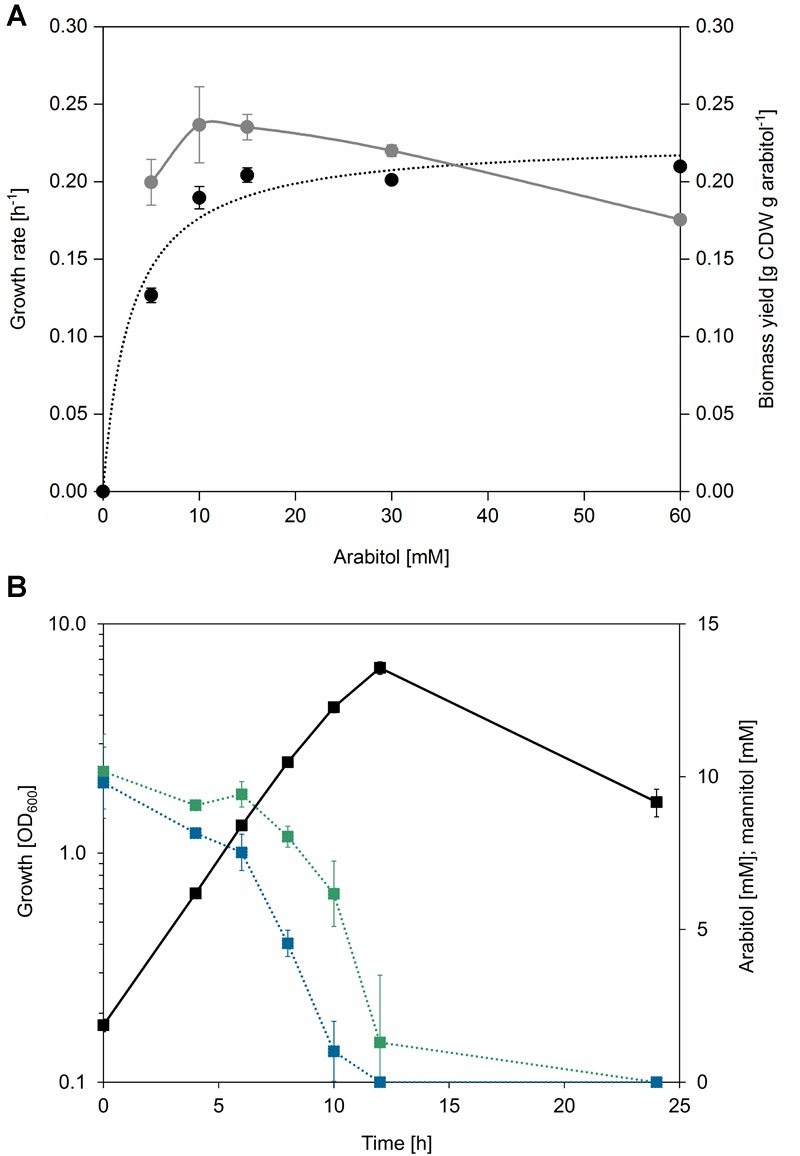
**(A)** Growth rates (black dots) and biomass yields (gray dots) of *B. methanolicus* MGA3 grown in minimal media containing 5, 10, 15, 30, and 60 mM arabitol. Full arabitol consumption was observed for all conditions except when 60 mM was used, in which case 19.1 ± 0.78 mM residual arabitol was detected after growth stopped. A relation between growth rate and substrate concentration was generated with the Michaelis Menten model using the OriginPro software version 2018 (OriginLab Corporation, Northampton, MA, United States). **(B)** Growth (black squares, solid lines) and residual substrate concentration (mannitol: blue squares, dotted lines; arabitol: green squares, dotted lines) of *B. methanolicus* MGA3 in minimal media containing a mixture of 15 mM mannitol and 15 mM arabitol. Mean values and standard deviations of triplicate shake flask cultures are given.

In search of gratuitous inducer of an *mtlR* promoter described by Irla et al. (2016), arabitol was tested as one of the potentially feasible compounds. While it was shown that addition of arabitol to cultivation broth does not lead to induction of expression of genes controlled by P*_mtlR_*, the authors have discovered that arabitol serves as sole carbon and energy source for *B. methanolicus* (Irla et al., 2016). Due to the fact that at that time only two alternative carbon sources had been described for this facultative methylotroph, it was interesting to investigate the physiology and genetic background of arabitol utilization in *B. methanolicus.* For that purpose, *B. methanolicus* MGA3 was cultivated in MVcM minimal media containing 10, 15, 30, or 60 mM arabitol. Arabitol was completely consumed in all tested conditions except when 60 mM was used, since about 20 mM remained in the supernatant when growth stopped (Supplementary Figure 1). Although arabitol supported growth of MGA3 as a sole source of carbon and energy, the strain grew at a lower growth rate than with its preferred sugar alcohol substrate mannitol: the growth rate using arabitol was 0.20 ± 0.01 h^-1^ as compared to 0.37 ± 0.01 h^-1^ when mannitol was used (Table 2). Accordingly, the substrate consumption rate was higher for mannitol than for arabitol (7.4 ± 0.5 vs. 5.7 ± 0.1 mmol g CDW^-1^ h^-1^), which was also the case for the biomass yield (0.28 ± 0.01 vs. 0.24 ± 0.01 g CDW g^-1^ or carbon normalized biomass yield 0.70 ± 0.02 vs. 0.60 ± 0.02 g CDW g carbon^-1^) (Table 2). As shown in Figure 2A, a relation between growth rate and substrate concentration according to Monod kinetics indicated that 2.9 ± 0.9 mM arabitol supported growth with a half-maximal growth rate.

**Table 2:** Growth rates, uptake rates, biomass yields, and other characteristics of *B. methanolicus* MGA3 grown on arabitol, mannitol, or a combination of both.

Carbon source(s) (15 mM each)	Growth rate (h^-1^)	Biomass yield (g CDW g^-1^)	Biomass yield (g CDW g carbon^-1^)	Uptake rate (mmol g CDW^-1^ h^-1^)
Arabitol	0.20 ± 0.01	0.24 ± 0.01	0.60 ± 0.02	5.7 ± 0.1
Mannitol	0.37 ± 0.01	0.28 ± 0.01	0.70 ± 0.02	7.4 ± 0.5
Arabitol + mannitol	0.31 ± 0.01	0.28 ± 0.01	0.70 ± 0.03	1.8 ± 0.0 (arabitol); 2.3 ± 0.1 (mannitol)

In order to test if arabitol and mannitol are utilized sequentially or simultaneously, growth of MGA3 in minimal media containing a mixture of 15 mM arabitol and 15 mM mannitol was analyzed (Figure 2B). MGA3 did not show biphasic growth in that experiment, and the maximum OD_600_ of 6.34 ± 0.41 was reached. Mannitol was utilized faster than arabitol and co-consumption of both sugar alcohols was observed between 6 and 12 h, at which point the growth stopped and both substrates were fully consumed (Figure 2B). As expected, the uptake rates for mannitol and arabitol during co-consumption (2.3 ± 0.1 and 1.8 ± 0.0 mmol g CDW^-1^ h^-1^, respectively) were lower than when either carbon source was provided as sole substrate (7.4 ± 0.5 and 5.7 ± 0.1 mmol g CDW^-1^ h^-1^, respectively) and the biomass yield using both mannitol and arabitol was the same as when only mannitol was provided to the media (0.28 ± 0.01 g CDW g^-1^) (Table 2).

### Comparative Analysis of Global Gene Expression Profiles of *B. methanolicus* MGA3 During Growth With Arabitol or Mannitol

In order to elucidate the genetic background of arabitol utilization, a differential gene expression analysis of *B. methanolicus* MGA3 cultivated with either 15 mM mannitol or 15 mM arabitol as sole carbon source was performed by RNA-seq. Sequencing of the prepared cDNA libraries from RNA isolated under the two chosen conditions resulted in 3,200,444 raw reads for cDNA library of mannitol grown cells and 2,728,707 raw reads for cDNA library of arabitol grown cells. Of these reads, 3,163,610 were mapped onto the genome of *B. methanolicus* MGA3 for the mannitol condition, leading to a coverage of 89.50% for the chromosome and 9.16 and 0.53% for the natural plasmids pBM19 and pBM69, respectively. For the arabitol condition, 2,684,972 reads were mapped onto the genome with an 88.16% coverage for the chromosome, 10.09% for pBM19 and 0.71% for pBM69 (Supplementary Table 2). During growth on arabitol, 48 genes showed significantly higher and 24 genes significantly lower RNA levels than during growth with mannitol (Supplementary Table 3). Of those, four genes involved in mannitol uptake and catabolism (Heggeset et al., 2012; Irla et al., 2016) and eight genes putatively involved in arabitol metabolism were selected and are shown in Table 3. As expected, genes coding for proteins related to mannitol metabolism showed higher RNA levels during growth on mannitol as compared to growth on arabitol. On the other hand, a gene cluster including genes BMMGA3_RS07325 to BMMGA3_RS07360 showed higher RNA levels during growth on arabitol as compared to mannitol-based growth (Table 3). The cluster comprises four genes annotated as coding for a PTS for galactitol uptake (BMMGA3_RS07330, BMMGA3_RS07335 and BMMGA3_RS07340) and a sorbitol dehydrogenase gene (BMMGA3_RS07345). However, neither galactitol nor sorbitol supported growth of *B. methanolicus* MGA3 (data not shown). Therefore, it was concluded that these genes may, in fact, be involved in arabitol metabolism.

**Table 3:** Key genes of mannitol and arabitol metabolism with altered expression in *B. methanolicus* MGA3 cultivated with arabitol in comparison to mannitol as sole carbon source.

Locus tag	Gene	Annotation	Log2 fold change of relative RNA levels (arabitol/ mannitol)^a^
BMMGA3_RS01065	*mtlA*^b^	PTS system mannitol-specific EIICB component^b^	-4.22
BMMGA3_RS01070	*mtlR*^b^	Transcriptional regulator MtlR^b^	-4.62
BMMGA3_RS01075	*mtlF*^b^	Mannitol-specific phosphotransferase enzyme IIA component^b^	-3.62
BMMGA3_RS01080	*mtlD*^b^	Mannitol-1-phosphate 5-dehydrogenase^b^	-3.87
BMMGA3_RS07325		Transcriptional antiterminator BglG	2.99
BMMGA3_RS07330	*atlA*^c^	IIA arabitol PTS component^c^	3.06
BMMGA3_RS07335	*atlB*^c^	IIB arabitol PTS component^c^	3.41
BMMGA3_RS07340	*atlC*^c^	IIC arabitol PTS component^c^	2.73
BMMGA3_RS07345	*atlD*^c^	Arabitol phosphate dehydrogenase^c^	2.90
BMMGA3_RS07350		Hypothetical protein	3.00
BMMGA3_RS07355		Galactitol-1-phosphate 5-dehydrogenase	2.97
BMMGA3_RS07360		*S*-methyl-5-thioribose-1-phosphate isomerase	2.15

*^*a*^Cut-off values set to a change in expression level higher than 30; *P* ≤ 0.01, determined by Student’s *t*-test. *^*b*^*Annotation according to Irla et al. (2016). ^*c*^Annotation according to this work’s findings.*

**FIGURE 3:**
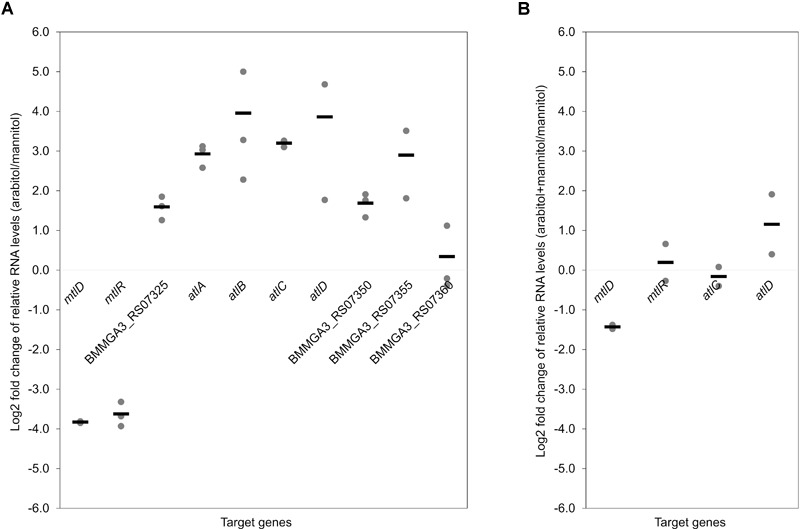
Dot plot graph depicts relative gene expression levels (shown in Log2 fold change) obtained in qRT-PCR of *B. methanolicus* MGA3 grown on arabitol in comparison to mannitol **(A)** and on a mixture of arabitol and mannitol in comparison to mannitol **(B)**. Graph represents individual data points and means as a bar.

To validate the RNA-seq results, qRT-PCR experiments were performed. As shown in Figure 3A, the expression levels of the targeted genes detected by qRT-PCR were in accordance with the gene expression patterns obtained by RNA-seq analysis (Table 3). Additionally, RNA levels of *mtlD, mtlR, atlC*, and *atlD* were determined by qRT-PCR during growth on the mixture of mannitol and arabitol as combined carbon sources. Differential expression was observed for genes *mtlD* and *atlD*, which code for catabolic enzymes, whereas RNA levels of the regulatory gene *mtlR* and the transport gene *atlC* did not change significantly (Figure 3B).

### Complementation of the Arabitol-Negative *C. glutamicum* Mutant Δ*mtlD* by Heterologous Expression of *B. methanolicus* MGA3 *atlABCD* or *atlD*

**FIGURE 4:**
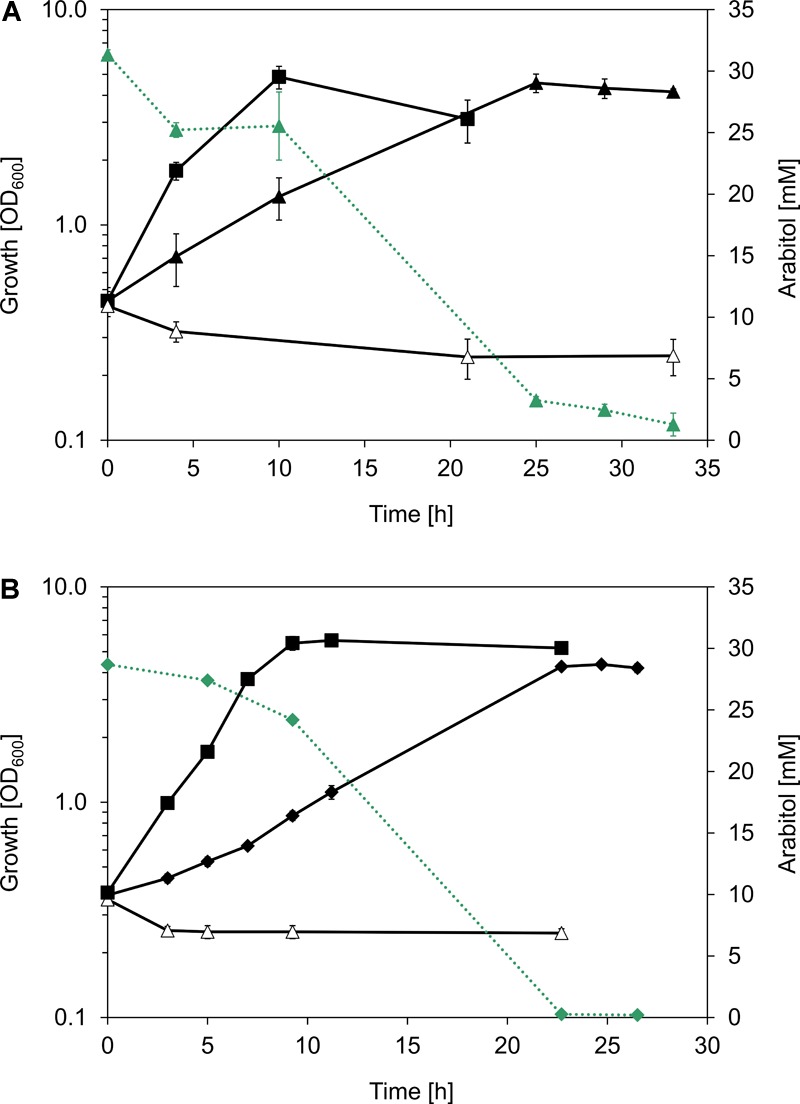
*B. methanolicus* MGA3 arabitol pathway genes complement an arabitol-negative *C. glutamicum* strain. **(A)** Growth (black, solid lines) of *C. glutamicum* strains WT(pVWEx1) (full squares), Δ*mtlD*(pVWEx1) (empty triangles), and Δ*mtlD*(pVWEx1-*atlABCD*) expressing *atlABCD* genes from *B. methanolicus* MGA3 under IPTG induction (full triangles) in minimal media containing 30 mM arabitol. **(B)** Growth (black, solid lines) of *C. glutamicum* strains WT(pVWEx1) (full squares), Δ*mtlD*(pVWEx1) (empty triangles) and Δ*mtlD*(pVWEx1-*atlD*) expressing the *atlD* gene from *B. methanolicus* MGA3 under IPTG induction (full diamonds) in minimal media containing 30 mM arabitol. Arabitol consumption (green, dotted lines) is depicted for *C. glutamicum* Δ*mtlD*(pVWEx1-*atlABCD*) and *C. glutamicum* Δ*mtlD*(pVWEx1-*atlD*). Mean values and standard deviations of triplicate shake flask cultures are given.

In order to verify the hypothesis that the *atlABCD* genes code for a PTS and an arabitol phosphate dehydrogenase and support arabitol catabolism, the arabitol-negative *C. glutamicum* Δ*mtlD* mutant (Laslo et al., 2012) was used for genetic complementation experiments. This experiment could not have been performed with an arabitol-negative *B. methanolicus* because gene deletion studies are currently not possible in this bacterium. *C. glutamicum* Δ*mtlD* is unable to grow with arabitol as sole carbon source (Laslo et al., 2012). Therefore, *atlABCD* genes from MGA3 were cloned into *C. glutamicum* expression vector pVWEx1, and the resulting vector pVWEx1-*atlABCD* was used to transform *C. glutamicum* Δ*mtlD* with the aim of restoring growth on arabitol. While the Δ*mtlD* mutant transformed with pVWEx1 empty vector was unable to grow on arabitol, heterologous expression of *B. methanolicus* MGA3-derived *atlABCD* genes from vector pVWEx1 allowed for growth with arabitol as sole carbon source and led to complete arabitol consumption (Figure 4A). However, since *C. glutamicum* Δ*mtlD* still possesses the arabitol transporter *rbtT* (Laslo et al., 2012), the possibility that the strain could still import arabitol via the native permease and that AtlD would subsequently take over its oxidation to xylulose could not be excluded. Therefore, we performed a complementation experiment in *C. glutamicum* Δ*mtlD* heterologously expressing the *B. methanolicus atlD* gene alone. Indeed, *atlD* could complement the deficiency of the Δ*mtlD* strain as efficiently as when the four *atlABCD* genes were heterologously expressed (Figure 4B and Supplementary Table 4). As expected, growth of *C. glutamicum* wild type (WT) with pVWEx1 empty vector on arabitol (Figure 4) and growth of *C. glutamicum* Δ*mtlD* on glucose was unaffected (Supplementary Figure 2). Thus, the *atlD* gene was shown to be sufficient to restore growth on arabitol of the arabitol-negative *C. glutamicum* Δ*mtlD* strain and provided evidence that *atlD* functions in arabitol utilization in *B. methanolicus* MGA3. Although the functionality of the *atlABC* genes could not be shown, their genetic organization and proximity to *atlD* in addition to the results obtained from the differential gene expression analysis strongly support their involvement in arabitol uptake, hence the genes were re-annotated to *atlABCD* (Table 3).

In an additional experiment, the *C. glutamicum* Δ*mtlD* mutant strain was complemented with the *atlABCD* and the two consecutive BMMGA3_RS07350 and BMMGA3_RS07355 genes. BMMGA3_RS07350 codes for a hypothetical protein and BMMGA3_RS07355 is annotated as coding for a galactitol-1-phosphate 5-dehydrogenase. However, growth and uptake rates weren’t significantly different than when only *atlD* or *atlABCD* from *B. methanolicus* MGA3 were used for complementation (Supplementary Table 4).

### Transcriptional Organization of the Arabitol Operon

**FIGURE 5:**
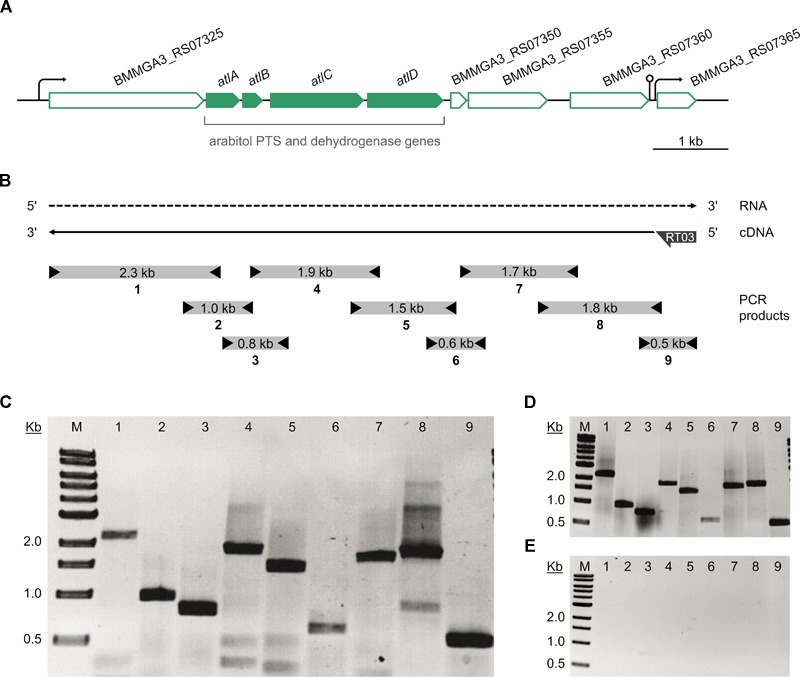
Transcriptional organization of the arabitol operon. **(A)** Genetic organization of the arabitol operon in *B. methanolicus* MGA3. Arrows represent the length and direction of the coding regions. **(B)** Representation of the RT-PCR analysis of the operon structure. The dashed arrow depicts the RNA transcript, the solid arrow depicts the cDNA generated by reverse transcription and subsequently used as template for PCR. Boxes with arrowheads depict the location and size of amplified PCR products (1–9) using primer pairs that spanned gene borders and the arrow at the cDNA 5′ end depicts the location of the gene specific primer RT03 hybridizing with BMMGA3_RS07365. **(C)** Agarose gel electrophoresis of the amplified PCR products using the RT-generated cDNA as template. **(D)** Agarose gel electrophoresis of the amplified PCR products using *B. methanolicus* MGA3 genomic DNA template as positive control. **(E)** Agarose gel electrophoresis of the amplified PCR products using no-RT (RNA) template as negative control. Lane M: 0.5–10 kb DNA ladder (NEB). Lanes 1 to 9: PCR products (1: 2,273 bp; 2: 958 bp; 3: 792 bp; 4: 1,858 bp; 5: 1,466 bp; 6: 629 bp; 7: 1,708 bp; 8: 1,794 bp; 9: 554 bp).

The *atlABCD* genes are clustered on the *B. methanolicus* genome and are arranged in the same transcriptional orientation as several neighboring genes (Figure 5A). Previous transcription analyses indicated that genes BMMGA3_RS07325 to BMMGA3_RS07355 might be organized in an operon (Irla et al., 2015). In addition to the previously described genes showing higher RNA levels during growth on arabitol in comparison to mannitol (Table 3), our RNA-seq data suggested that BMMGA3_RS07365 might also be co-transcribed with the preceding genes based on reads spanning two genes. For independent confirmation of that assumption, RT-PCR experiments were performed. RNA obtained during growth on arabitol was reverse transcribed with primer RT03 annealing downstream of BMMGA3_RS07365 and used as template for PCRs with primer pairs spanning the gene borders (Figure 5B). The observed amplification products indicated that indeed genes BMMGA3_RS07325 to BMMGA3_RS07365 are co-transcribed as an operon (Figure 5C). As positive control, *B. methanolicus* MGA3 genomic DNA was used as template (Figure 5D). The absence of contaminations of the RNA preparation with genomic DNA was confirmed in PCRs using RNA as template (Figure 5E). Thus, genes *atlA, atlB, atlC*, and *atlD* are part of a larger operon that ranges from BMMGA3_RS07325 to BMMGA3_RS07365.

### Arabitol Phosphate Dehydrogenase Activity of *B. methanolicus* MGA3 Crude Extracts

In order to confirm whether *B. methanolicus* MGA3 possesses an arabitol phosphate dehydrogenase and to assay if its activity is increased during growth with arabitol, crude extracts of MGA3 cells grown on arabitol, mannitol or methanol were prepared and arabitol phosphate dehydrogenase activities were determined. The enzyme assays were carried as described by Povelainen et al. (2003) with xylulose 5-phosphate as substrate. Since arabitol 1-phosphate and arabitol 5-phosphate were not available, arabitol phosphate oxidation could not be assayed. Instead, reduction of xylulose 5-phosphate was assayed and arabitol phosphate dehydrogenase activity was shown in *B. methanolicus* crude extracts. As expected, the highest enzyme activity (0.05 ± 0.01 U mg^-1^) was detected in extracts of cells grown on arabitol (Table 4). Surprisingly, mannitol grown cells showed, albeit reduced, arabitol phosphate dehydrogenase activity (0.02 ± 0.00 U mg^-1^), while methanol grown cells lacked detectable arabitol phosphate dehydrogenase activity (Table 4). Using the crude extracts prepared from arabitol grown cells, the *K*_M_ value for the substrate xylulose 5-phosphate was determined to be 0.03 ± 0.02 mM. Although this is a rough estimate obtained with crude extracts rather than with the purified enzyme, the sub-millimolar *K*_M_ value indicates high affinity of the arabitol phosphate dehydrogenase from *B. methanolicus* for the substrate xylulose 5-phosphate and is in line with the arabitol concentration supporting half-maximal growth (*K*_S_ of 1.2 ± 0.3 mM).

**Table 4:** Specific activities of arabitol phosphate dehydrogenase in cell extracts of *B. methanolicus* MGA3 grown in minimal medium containing different carbon sources.

Carbon source	Mean specific activity (U mg^-1^)
Arabitol	0.05 ± 0.01
Mannitol	0.02 ± 0.00
Methanol	<0.01

## Discussion

**FIGURE 6:**
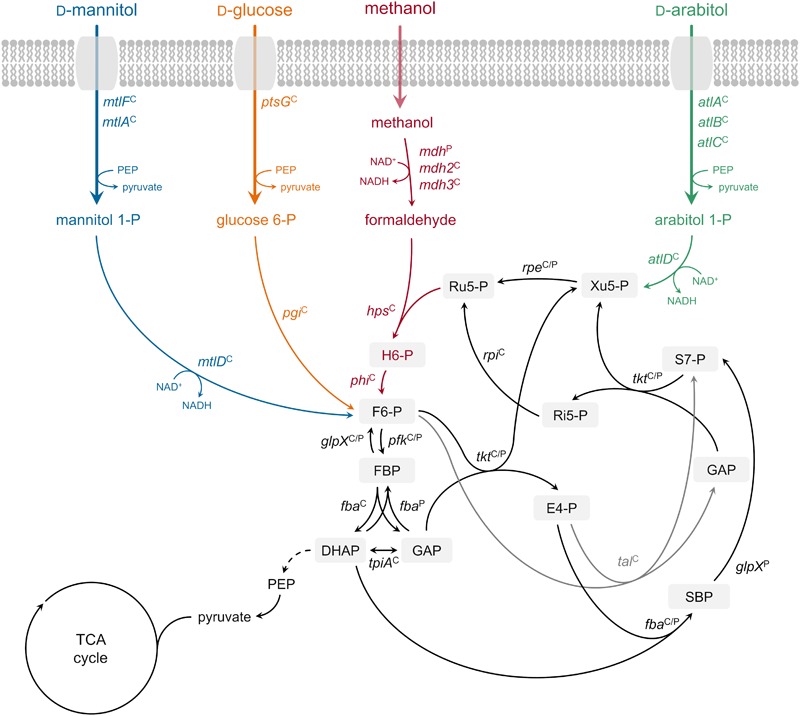
Ribulose monophosphate cycle and proposed methylotrophic and carbohydrate assimilation pathways in *B. methanolicus* MGA3. Genes: *mdh*, methanol dehydrogenase (EC 1.1.1.244); *hps*, 3-hexulose-6-phosphate synthase (EC 4.1.2.43); *phi*, 6-phospho-3-hexuloisomerase (EC 5.3.1.27); *pfk*, 6-phosphofructokinase, (EC 2.7.1.11); *fba*, fructose-bisphosphate aldolase (EC 4.1.2.13); *tkt*, transketolase (EC 2.2.1.1); *glpX*, fructose-bisphosphatase (EC 3.1.3.1); *tal*, transaldolase (EC 2.2.1.2); *rpe*, ribulosephosphate 3-epimerase (EC 5.1.3.1); *rpi*, ribose-5-phosphate isomerase (EC 5.3.1.6); *tpiA*, triosephosphate isomerase (EC 5.3.1.1); *ptsG*, PTS-glucose-specific transporter subunit IICBA (EC 2.7.1.69) *pgi*, glucose-6-phosphate isomerase (EC 5.3.1.9); *mtlF*, mannitol-specific phosphotransferase enzyme IIA component (EC 2.7.1.69); *mtlA*, PTS system mannitol-specific enzyme IIBC component (EC 2.7.1.69); *mtlD*, mannitol-1-phosphate 5-dehydrogenase (EC 1.1.1.17); *atlA*, IIA arabitol PTS component; *atlB*, IIB arabitol PTS component; *atlC*, IIC arabitol PTS component; *atlD*, arabitol phosphate dehydrogenase. Metabolites: H6-P, 3-hexulose 6-phosphate; F6-P, fructose 6-phosphate; FBP, fructose 1,6-bisphosphate; GAP, glyceraldehyde 3-phosphate; DHAP, dihydroxyacetone phosphate; E4-P, erythrose 4-phosphate; SBP, sedoheptulose 1,7-bisphosphate; S7-P, sedoheptulose 7-phosphate; Ri5-P, ribose 5-phosphate; Xu5-P, xylulose 5-phosphate; Ru5-P, ribulose 5-phosphate; PEP, phosphoenolpyruvate; TCA tricarboxylic acid. Superscript “C”: chromosomally encoded; superscript “P”: natural plasmid pBM19 encoded. Phosphoenolpyruvate–protein phosphotransferase [enzyme EI (EC 2.7.3.9)] and phosphocarrier protein (HPr) are not depicted.

Growth of *B. methanolicus* MGA3 on the sugar alcohol D-arabitol is characterized for the first time. Based on an RNA-seq analysis of global gene expression during growth on this sugar alcohol, genetic and biochemical investigation of a role of *atlABCD* encoding a PTS and arabitol phosphate dehydrogenase in uptake and activation of arabitol is demonstrated. Figure 6 depicts the proposed pathway for arabitol uptake and catabolism next to the known pathways for methanol, mannitol and glucose utilization operating in *B. methanolicus* MGA3.

The finding that *B. methanolicus* MGA3 is able to catabolize low arabitol concentrations effectively (*K*_S_ value of about 3 mM) is in line with our prediction that arabitol is taken up by the cells via arabitol PTS encoded by arabitol inducible genes *atlABC*. High substrate affinity has been reported in several organisms in relation to PTS-mediated uptake (Nothaft et al., 2003; Lindner et al., 2011; Opačić et al., 2012), as is also the case for mannitol in *B. methanolicus*: the *K*_S_ value for mannitol was determined to be 0.2 ± 0.1 mM with genes *mtlF, mtlA*, and *mtlD* known to code for a mannitol-specific PTS enzyme IIA component, a mannitol-specific PTS enzyme IIBC component and a mannitol-1-phosphate 5-dehydrogenase, respectively (Heggeset et al., 2012; Irla et al., 2016). However, the observed growth rates and substrate uptake rates were higher for mannitol than for arabitol, and the biomass yield for the C5 sugar alcohol arabitol was lower than for the C6 sugar alcohol mannitol (Table 2). Methanol allows growth rate and biomass yield similar to those observed for mannitol-based growth (Jakobsen et al., 2006; Nilasari et al., 2012). Thus, if a mixture of methanol, mannitol and arabitol is present in the natural habitat, arabitol is expected to contribute to growth of *B. methanolicus* albeit slower and less efficient as compared to methanol and mannitol. This is likely even more pronounced if high arabitol concentrations are encountered in its ecological niche, since *B. methanolicus* cannot utilize arabitol efficiently at concentrations exceeding 30 mM, as biomass yields with 60 mM arabitol, for example, were only moderately higher than with 30 mM and residual arabitol was observed when growth stopped (Supplementary Figure 1).

Co-consumption of arabitol with mannitol and monophasic growth were observed with an equimolar mixture of both sugar alcohols as combined carbon source (Table 2). Monophasic growth with simultaneous substrate consumption has been previously reported for e.g., *C. glutamicum* in a mixture of glucose and pyruvate (Cocaign et al., 1993), glucose and fructose (Dominguez et al., 1997) or glucose and acetate (Wendisch et al., 2000), *Mycobacterium tuberculosis* in a mixture of glucose, acetate, and/or glycerol (de Carvalho et al., 2010) and *Bacillus subtilis* in a mixture of glucose and malate (Kleijn et al., 2010), although consumption of single carbon substrates in a preferred order displaying diauxic growth, consequence of catabolite repression, is a more widespread mechanism in most bacteria (Kovárová-Kovar and Egli, 1998). The fact that both arabitol and mannitol uptake rates were more than three-fold lower during growth with the mixture than with either sugar alcohol alone (Table 2) indicated regulation of carbon source utilization. This assumption is confirmed by our qRT-PCR analysis (Figure 3B) where *mtlD* transcript levels are lower in the cells cultivated on mixture of arabitol and mannitol in comparison to mannitol only. Inhibition of glucose uptake during growth on glucose in the presence of arabitol was previously reported by Laslo et al. (2012) both for the wild type and the *C. glutamicum* Δ*mtlD* mutant strain. Co-consumption of glucose and xylose led, too, to lower consumption rates for glucose in the *E. coli* Δ*ptsG* mutant (Matsuoka and Shimizu, 2013), while glucose was shown to inhibit pentose uptake in *Saccharomyces cerevisiae* (Subtil and Boles, 2012).

Differential gene expression analysis of *B. methanolicus* MGA3 grown on arabitol as compared to mannitol detected higher RNA levels for *mtlA, mtlR, mtlF*, and *mtlD* in mannitol-grown cells (Table 3) as expected (Heggeset et al., 2012; Müller et al., 2014; Irla et al., 2016). The higher RNA levels observed for the putative arabitol PTS genes *atlA, atlB*, and *atlC* as well as arabitol phosphate dehydrogenase gene *atlD* during growth on arabitol compared to growth with mannitol might be due to arabitol induction or lack of mannitol repression. In *C. glutamicum*, arabitol induces expression of *rbtT, mtlD, sixA, xylB* and *atlR* and glucose represses expression of *mtlD* (Laslo et al., 2012). In *B. methanolicus*, RNA levels for *atlD* were higher while lower for *mtlD* during growth with both carbon sources as compared to growth with mannitol alone (Figure 3B). By contrast, the RNA levels of both *mtlR* and *atlC* were comparable under both conditions. This may indicate arabitol repression of *mtlR* and mannitol repression of *atlC* in the arabitol/mannitol mixture condition. Moreover, this observation is in line with the finding that both carbon sources are utilized faster when present alone as compared to growth with a mixture of mannitol and arabitol (Table 2). Contrarily, in *Pseudomonas fluorescens* DSM 50106 mannitol, arabitol and glucitol are inducers for transcription of *mtl* operon coding for proteins involved in transport and utilization of those sugar alcohols and the gene expression is regulated by the transcriptional regulator MtlR (Hoffmann and Altenbuchner, 2015).

Here, the physiological role of the *altABCD* genes in the utilization of arabitol by *B. methanolicus* was analyzed via complementation studies. Growth with arabitol of the arabitol growth-deficient *C. glutamicum* Δ*mtlD* strain was restored by heterologous expression of *atlABCD* as well as *atlD* from *B. methanolicus*. However, involvement of *atlABC* in arabitol uptake in *B. methanolicus* could not be confirmed via complementation experiments: the phenotypes of *C. glutamicum* Δ*mtlD*(pVWEx1-*atlABCD*) and *C. glutamicum* Δ*mtlD*(pVWEx1-*atlD*) did not show significant differences (Figure 4 and Supplementary Table 4). The fact that the *atlABC* genes were not required for arabitol uptake in the Δ*mtlD* mutant was additionally supported by the displayed substrate affinity: the arabitol affinity of the PTS in *B. methanolicus* was estimated at the level of 2.9 ± 0.9 mM, whereas the arabitol *K*_S_ for *C. glutamicum* Δ*mtlD*(pVWEx1-*atlABCD*) was almost ten-fold higher, namely 9.4 ± 0.3 mM (Supplementary Figure 3A). This result is similar to arabitol *K*_S_ in *C. glutamicum* WT(pVWEx1) of 8.3 ± 2.0 mM (Supplementary Figure 3B) or wild type *C. glutamicum* 7.91 ± 0.52 mM (Laslo et al., 2012). The difference of *K*_S_ of *B. methanolicus*-derived PTS system in the genetic background of *B. methanolicus* and *C. glutamicum* Δ*mtlD* might be due to the presence of the native arabitol permease *rbtT* in the latter. The observation that only *atlD* was necessary to complement the *C. glutamicum* Δ*mtlD* deficiency implies that either AtlD has arabitol dehydrogenase activity besides the here determined arabitol phosphate dehydrogenase activity (Table 4) or that *C. glutamicum* can import arabitol via an additional uptake route, which would be supported by the fact that *C. glutamicum* Δ*rbtT*, although poorly, can still grow on arabitol (Laslo et al., 2012). Despite the fact that the *atlABC* genes were not found necessary to complement *C. glutamicum* Δ*mtlD*, their participation in arabitol uptake in *B. methanolicus* cannot be excluded. As seen from the differential gene expression analysis, *atlABCD* were clearly up-regulated under arabitol conditions (Table 3). Moreover, transcriptional organization experiments revealed that said genes are part of the same operon and the genetic organization is in accordance to previously reported arabitol PTS and dehydrogenase genes (Povelainen et al., 2003; Kentache et al., 2016). BLASTp analyses recognized AtlABC as part of the multienzyme PTS complex involved in the transport and phosphorylation of carbohydrates. The PTS phosphorylation cascade involves the general PTS components phosphoenolpyruvate–protein phosphotransferase enzyme I (EI) and phosphocarrier protein (HPr), and the carbohydrate-specific permease enzyme II, consisting of two cytoplasmic domains (IIA and IIB) and a transmembrane channel domain (IIC, with or without IID depending on the system) (Saier, 2015; Kentache et al., 2016). Homology comparisons identified AtlA as PTS sugar transporter subunit IIA inside the family of fructose/mannitol specific IIA subunit enzymes (cd00211), AtlB as subunit IIB of enzyme II of the galactitol-specific PTS (cd05566) and AtlC as PTS galactitol-specific IIC component (COG3775). EI autophosphorylates using phosphoenolpyruvate (PEP) as phosphoryl donor, which in turn transfers the phosphoryl group to HPr (Saier, 2015; Kentache et al., 2016). Following this, we propose that HPr phosphorylates arabitol-specific EIIA AtlA, which subsequently transfers the phosphoryl group to EIIB component AtlB and, in the last step, donates its phosphoryl group to arabitol bound to EIIC transmembrane domain AtlC, releasing arabitol-phosphate into the cytoplasm. Sequence comparison of characterized transmembrane permease IIC for the arabitol AltC from *L. monocytogenes* (Saklani-Jusforgues et al., 2001) with *B. methanolicus* AtlC showed identity at the level of 56%.

Furthermore, the comparison of the sequence of the putative arabitol phosphate dehydrogenase encoded by *altD* gene with an amino acid sequence of characterized arabitol 1-phosphate dehydrogenase from *E. avium* (Povelainen et al., 2003) showed 51% identity between these proteins. Arabitol phosphate dehydrogenase (AtlD) activities were tested in crude extracts of MGA3 cells grown on arabitol, mannitol or methanol (Table 4). The results confirm that *B. methanolicus* MGA3 indeed possesses an arabitol phosphate dehydrogenase with increased activity during growth with arabitol, which is in accordance with the differential gene expression and the qRT-PCR analyses comparing arabitol- to mannitol-based growth (Table 3 and Figure 3). Additionally, the *K*_M_ value for xylulose 5-phosphate was determined to be 0.03 ± 0.02 mM using crude extracts from cells grown on arabitol, which shows high substrate affinity (Supplementary Figure 4). Despite the fact that the reaction was not assayed in the physiological direction (i.e., oxidation of arabitol 1-phosphate or arabitol 5-phosphate to xylulose 5-phosphate), the enzyme kinetics are in accordance with the *K*_S_ value of 2.9 ± 0.9 mM determined for arabitol. Although AtlD affinity for the substrate xylulose 5-phosphate was not determined using the purified enzyme, our results are in the range of previously characterized arabitol phosphate dehydrogenase in *E. avium* (Povelainen et al., 2003). Taken all together, these results strongly indicate the functionality of the *altABCD* encoded proteins in the arabitol utilization in *B. methanolicus* MGA3.

Addition of arabitol to cultivation broth not only induced expression of *atlABCD*, but also of adjacent genes, i.e., eight genes from BMMGA3_RS07325 to BMMGA3_RS07360 and all part of the arabitol operon as established via RT-PCR analysis (Figure 5). BMMGA3_RS07325 codes for a putative transcriptional antiterminator BglG. The *bgl-sac* family of antiterminator proteins are effectors of substrate-induced antitermination of catabolic operons and include, e.g., SacT and SacY of *B. subtilis* (Debarbouille et al., 1990; Manival, 1997) and BglG of *E. coli* (Nussbaum-Shochat and Amster-Choder, 1999; Raveh et al., 2009). These antiterminator proteins have been extensively characterized and act as RNA-binding proteins abrogating termination of transcription and allowing transcription elongation (Rutberg, 1997; Van Assche et al., 2015). A BLASTp analysis showed that the protein encoded by BMMGA3_RS07325 shared 30% identity to BglG of *E. coli*, 35% identity to SacY and 37% identity to SacT of *B. subtilis*, and 40% identity to BglG of *L. monocytogenes* (Gorski et al., 2003). Additionally, both *E. coli bglG* and *B. subtilis sacT* are located upstream of genes coding for PTS components involved in the utilization of ß-glucosides and sucrose, respectively (Rutberg, 1997). Similar gene order, where gene coding for antiterminator is upstream of genes encoding arabitol PTS, was observed in an arabitol gene cluster in *L. monocytogenes* and *E. avium* (Gorski et al., 2003; Povelainen et al., 2003). Interestingly, the other *B. methanolicus* wild type strain PB1 (NCIMB13113) is unable to grow on arabitol (data not shown). Both strains have been previously reported to show physiological differences (Heggeset et al., 2012). A BLAST analysis comparing the arabitol operon sequences of MGA3 and PB1 showed an incomplete BMMGA3_RS07325 gene sequence in the genome of the PB1 strain. These findings may indicate that BMMGA3_RS07325 functions as regulator of arabitol catabolism in *B. methanolicus* MGA3, and that its truncated form in *B. methanolicus* PB1 leads to absence of growth on arabitol.

RT-PCR and RNA-seq analysis revealed that genes BMMGA3_RS07325 to BMMGA3_RS07365 are co-transcribed as an operon (Figure 5). Accordingly, conserved promoter motifs were present upstream of BMMGA3_RS07325 (Irla et al., 2015). Using the ARNold tool for identification of transcriptional terminators (Naville et al., 2011), additional promoter motifs were found between BMMGA3_RS07360 and BMMGA3_RS07365 overlapping with a putative terminator structure (data not shown). These findings suggest the presence of a sub-operon starting at BMMGA3_RS07365 that would most likely not be involved in arabitol metabolism since it is not induced by arabitol. It is therefore remarkable that BMMGA3_RS07365 is co-transcribed alongside the arabitol inducible genes.

*Bacillus methanolicus* MGA3 is a facultative methyloptroph with a narrow substrate spectrum. Here, we have identified and characterized growth with D-arabitol. RNA-seq analysis revealed evidence for arabitol inducible catabolism of this sugar alcohol via a PTS AtlABC and an arabitol phosphate dehydrogenase AtlD, and genetic complementation studies confirmed functionality of the latter during arabitol metabolism. The role of a second putative arabitol phosphate dehydrogenase co-transcribed with *atlABCD* and metabolic fluxes during growth with arabitol remain to be studied. Once established for *B. methanolicus*, gene deletion experiments combined with biochemical characterization of the enzymes and ^13^C labeling experiments will help to further our understanding on how this sugar alcohol is catabolized as sole or combined carbon source.

## Data Availability

All datasets generated for this study are included in the manuscript and/or the Supplementary Files. The data sets supporting the results of this article are available in the NCBI Gene Expression Omnibus database under the accession number GSE133849, https://www.ncbi.nlm.nih.gov/geo/query/acc.cgi?acc=GSE133849.

## Author Contributions

ML carried out the experimental procedures. ML, MI, and LB analyzed the data. ML prepared a draft of the manuscript. ML, MI, LB, and VW finalized the manuscript. VW coordinated the study. All authors read and approved the final version of the manuscript.

## Conflict of Interest Statement

The authors declare that the research was conducted in the absence of any commercial or financial relationships that could be construed as a potential conflict of interest.
