# Supplementary material for: Characterization of D-Arabitol as Newly Discovered Carbon Source of Bacillus methanolicus
Source: Front Microbiol. 2019 Jul 31;10:1725. doi: 10.3389/fmicb.2019.01725 (PMC6685057; doi:10.3389/fmicb.2019.01725)
Supplement: Supplementary file 1 [file Data_Sheet_1.pdf]

Supplementary Material

Supplementary Tables

Supplementary Table 1. Primer sequences used in this study.

Primer	Sequence [5' → 3']	Characteristics
acF1	cctgcaggtcgactctagaggaa**aggagg**cccttcag GTGAGTGAAATATACTTTGATGA ATC	Amplification of *atlABCD* and *atlABCDEF* for pVWEx1-*atlABCD* and pVWEx1-*atlABCDEF* (fw)
acR1	attcgagctcggtacccgggTTAATCAATAG GTGTCAACAATAC	Amplification of *atlABCD* for pVWEx1-*atlABCD* (rv)
acR2	attcgagctcggtacccgggTTATGATTTTT CTGGATGGAAG	Amplification of *atlABCDEF* for pVWEx1-*atlABCDEF* (rv)
aPDF	cctgcaggtcgactctagaggaa**aggagg**cccttcag ATGAAAGCTTTAGTCAAAAAAG	Amplification of *atlD* for pVWEx1-*atlD*
aPDR	gagctcggtacccggggatcTTAATCAATA GGTGTCAACAATAC	
MRF1	TACATAAATAGGAGGTAGTAcatA TGTATATATCTGCAAGAGAAAG	Amplification of *mtlR* to confirm the absence of gDNA contamination of the RNA preparation
MRR1	TGGCGGGTACCATATGGATCCTT ATACACTCCTTAATTCTTTTAATT TTTC	
PRIF	GAAAGGAGGCCCTTCAGATGAAG AAGCTTACTTTTGTCGGAGCAGGT	Amplification of *proI* to confirm the absence of gDNA contamination of the RNA preparation
PRIR	TGGCGGGTACCATATGGATCCTC ACTGCTTTACTGTTACTGCTTCTG TGT	
RT03	ATAGAATCGGAATAGTCATACCC ATCTC	BMMGA3_RS07365-BMMGA3_RS07325 cDNA synthesis
1fwd	ATGATGCTTGATGTCAGATGTG	Amplification of BMMGA3_RS07325 for transcriptional organization analysis
2rvs	GAAATGCCATTTGTAGGCAAAC	
3fwd	GTCATTGCTCCGAATACAGCGATT CCTC	Amplification of BMMGA3_RS07330 (*atlA*) for transcriptional organization analysis
4rvs	ACAGATCGGCCGTATCGACATAT GAACC	
5fwd	GCGACTGGTTTGCCTACAAATG	Amplification of BMMGA3_RS07335 (*atlB*) for transcriptional organization analysis
6rvs	GCCAAACAGCAAGCCTAGTATG	
7fwd	GCAAAGCGATAGAAGACC	Amplification of BMMGA3_RS07340 (*atlC*) for transcriptional organization analysis
8rvs	CTGACGTTACTCTGTCTC	
9fwd	TGTCGCATTTATCGTCGGTGCTTC	Amplification of BMMGA3_RS07345 (*atlD*) for transcriptional organization analysis
10rv	CCTATCATTCTCCCATACGGTCCA AGTG	
11fw	ACGATGGTCGGATCTAGAAG	

12rv	CAGAGCCGCAAATACCTG	Amplification of BMMGA3_RS07350 for transcriptional organization analysis
13fo	GCACTTGGACCGTATGGGAGAATG	Amplification of BMMGA3_RS07355 for transcriptional organization analysis
14ro	AATTGCCGGTGCTCCTCGAAC	
15fo	GATCTCAGCTCCATTTCC	Amplification of BMMGA3_RS07360 for transcriptional organization analysis
16ro	AGTCAGGTGCTTTCACTC	
17fw	GCCTTTGATGTAACACCTCATGAACTC	Amplification of BMMGA3_RS07365 for transcriptional organization analysis
18rv	AACCGTTGAACATCATATTGCGGATCG	
P151	TGGGAGTAGTGGAACAGAAG	qRT-PCR analysis of *repB* expression
P152	GCCAAGCAACCTGTATTACC	
P135	ATTTGCTCAAGCGGCATAGG	qRT-PCR analysis of *mtlR* expression
P136	GTTAGGCAGGTTTACCGTAG	
P139	AACAAGGGTGGCCGTTCTC	qRT-PCR analysis of *mtlD* expression
P140	ACCGCTTCCGCATCTTGTGG	
P141	CCCATTGGTTGGATGGTTTC	qRT-PCR analysis of *phi* expression
P142	AATCATTGGATCCGGCTCAG	
P143	CAAGATCCGGTTCTGCTTTG	qRT-PCR analysis of *hps* expression
P144	TGGATGAAATGGGCGTAGAC	
P158	TGATTACATTGCGCCACTCG	qRT-PCR analysis of BMMGA3_RS07325 expression
P159	GTGAGGAATCGCTGTATTCG	
P160	ATCGACGCTGTCATTGAGAG	qRT-PCR analysis of BMMGA3_RS07330 (*atlA*) expression
4qrv	ACGCCAAATTTCACGGGTTC	
P162	AGCCTGCGGAACAGGAATTG	qRT-PCR analysis of BMMGA3_RS07335 (*atlB*) expression
P163	CTAACAGATCGGCCGTATCG	
P164	GGAATCGGCACGTGAATTAC	qRT-PCR analysis of BMMGA3_RS07340 (*atlC*) expression
P165	CGTTGCTAAGTCACCGAATG	
P166	TTAGGTCCCGGACCAATAGG	qRT-PCR analysis of BMMGA3_RS07345 (*atlD*) expression
P167	ACATCAGCACCGTATCCATC	
P168	TTGGACCGTATGGGAGAATG	qRT-PCR analysis of BMMGA3_RS07350 expression
P169	TTAACGCTTATGGCCTCCTG	
P160	TCAGCCGGTTCTCCTGTTAC	qRT-PCR analysis of BMMGA3_RS07355 expression
P171	GAGATCGCATTCCAAGATCC	
P145	TTGGCAACATCAAGGCCAAC	qRT-PCR analysis of BMMGA3_RS07360 expression
P146	TCTTCGTGGCGGATTAAGTG	
P172	TTGCAAGCAGGAGTGAAAGC	qRT-PCR analysis of BMMGA3_RS07365 expression
P173	ACATTCACTGCCAGGATGAC	

Overlapping regions are shown in lower case, ribosome binding sites are shown in bold; fw: forward primer, rv: reverse primer.

Supplementary Table 2. Mapping and coverage characteristics of *B. methanolicus* MGA3 generated cDNA libraries.

		Mannitol condition	Arabitol condition
Raw reads		3,200,444	2,728,707
Mapped reads		3,163,610	2,684,972
Coverage	Chromosome	89.50 %	88.16 %
	Natural plasmid pBM19	9.16 %	10.09 %
	Natural plasmid pBM69	0.53 %	0.71 %

Supplementary Table 3. List of genes with altered expression in *B. methanolicus* MGA3 cultivated with arabitol in comparison to mannitol as sole carbon source.

Locus tag	Gene	Annotation	Log2 fold change of relative RNA levels (arabitol/ mannitol)[a]
**BMMGA3_RS01065**	***mtlA*[b]**	**PTS mannitol transporter subunit IICBA[b]**	**-4.2**
**BMMGA3_RS01070**	***mtlR*[b]**	**transcriptional regulator MtlR[b]**	**-4.6**
**BMMGA3_RS01075**	***mtlF*[b]**	**PTS mannitol transporter subunit IIA[b]**	**-3.6**
**BMMGA3_RS01080**	***mtlD*[b]**	**mannitol-1-phosphate 5-dehydrogenase[b]**	**-3.9**
BMMGA3_RS01710		adenine deaminase	2.3
BMMGA3_RS02480		sigma-54-dependent Fis family transcriptional regulator	3.2
BMMGA3_RS16990		*inner spore coat protein D (CotD)*	5.3
BMMGA3_RS03910		long-chain-fatty-acid-CoA ligase	2.2
BMMGA3_RS04600	*glnH*	glutamine ABC transporter substrate-binding protein	2.1
BMMGA3_RS04630		methyl-accepting chemotaxis protein	-5.0
BMMGA3_RS04635		thioester reductase	-2.8
BMMGA3_RS04655		PilZ domain-containing protein	-4.5
BMMGA3_RS04675		*No putative conserved domains*	-3.2
BMMGA3_RS04745	*ytdA*	UTP--glucose-1-phosphate uridylyltransferase	3.8
BMMGA3_RS05030		*YkyB-like protein*	2.4
BMMGA3_RS05290		cytochrome c oxidase subunit IVB	-2.4
BMMGA3_RS05610	*pyrP*	uracil transporter	-2.3
BMMGA3_RS05615	*pyrB*	aspartate carbamoyltransferase catalytic subunit	-3.1

BMMGA3_RS05625	*pyrAA*	carbamoyl-phosphate synthase small subunit	-2.0
BMMGA3_RS05645	*pyrF*	orotidine-5'-phosphate decarboxylase	-2.0
BMMGA3_RS06870		*No putative conserved domains*	4.1
BMMGA3_RS06885	*gca*	GDP-mannose 4,6-dehydratase	3.3
BMMGA3_RS06910		*No putative conserved domains*	5.8
BMMGA3_RS06940		Spore coat protein	4.9
BMMGA3_RS07255		*No putative conserved domains*	6.3
**BMMGA3_RS07325**		**transcriptional antiterminator BglG**	**3.0**
**BMMGA3_RS07330**	***atlA*[c]**	**IIA arabitol PTS component[c]**	**3.1**
**BMMGA3_RS07335**	***atlB*[c]**	**IIB arabitol PTS component[c]**	**3.4**
**BMMGA3_RS07340**	***atlC*[c]**	**IIC arabitol PTS component[c]**	**2.7**
**BMMGA3_RS07345**	***atlD*[c]**	**Arabitol phosphate dehydrogenase[c]**	**2.9**
**BMMGA3_RS07350**		***No putative conserved domains***	**3.0**
**BMMGA3_RS07355**		**galactitol-1-phosphate 5-dehydrogenase**	**3.0**
**BMMGA3_RS07360**	***mtnA***	**S-methyl-5-thioribose-1-phosphate isomerase**	**2.2**
BMMGA3_RS07750	*desR*	DNA-binding response regulator	-3.8
BMMGA3_RS07755	*desK*	sensor histidine kinase	-4.0
BMMGA3_RS07760	*des*	fatty acid desaturase	-5.5
BMMGA3_RS07780		glutamine amidotransferase	3.5
BMMGA3_RS07980		molybdopterin molybdenumtransferase MoeA	2.2
BMMGA3_RS07985		nucleotidyltransferase family protein	2.6
BMMGA3_RS07990		xanthine dehydrogenase	2.6
BMMGA3_RS08000		VWA domain-containing protein	3.1
BMMGA3_RS08005		MoxR family ATPase	3.0
BMMGA3_RS08010		carbon monoxide dehydrogenase subunit G	3.3
BMMGA3_RS08015		YHS domain-containing protein	3.5
BMMGA3_RS08020	*cutL*	carbon-monoxide dehydrogenase large subunit	3.5
BMMGA3_RS08025	*cutS*	(2Fe-2S)-binding protein	3.1
BMMGA3_RS08030		molybdopterin dehydrogenase FAD-binding protein	3.4
BMMGA3_RS08045	*dhaS2*	xanthine dehydrogenase family protein subunit M	3.4
BMMGA3_RS08220		*amyloid fiber anchoring/assembly protein TapA*	2.1
BMMGA3_RS10055		*No putative conserved domains*	-4.6
BMMGA3_RS10715	*scoB*	CoA transferase subunit B	2.8
BMMGA3_RS10720	*scoA*	CoA transferase subunit A	2.6
BMMGA3_RS10740		cytochrome ubiquinol oxidase subunit I	-2.5
BMMGA3_RS12425	*lcfA*	long-chain fatty acid--CoA ligase	2.4
BMMGA3_RS13000		DeoR family transcriptional regulator	-3.8

BMMGA3_RS13035	*ywcA*	cation acetate symporter	2.1
BMMGA3_RS13925	*fadE*	acyl-CoA dehydrogenase	2.8
BMMGA3_RS13930	*fadA*	acetyl-CoA acetyltransferase	2.3
BMMGA3_RS13935	*fadN*	3-hydroxyacyl-CoA dehydrogenase	2.8
BMMGA3_RS14465		flagella export chaperone FliS	-2.6
BMMGA3_RS14470	*fliD*	flagellar cap protein FliD	-2.6
BMMGA3_RS14490		flagellin domain protein	-2.9
BMMGA3_RS14735		*No putative conserved domains*	-2.4
BMMGA3_RS15110		DNA-binding protein	-2.6
BMMGA3_RS15420	*nuoD*	NAD(P)H-quinone oxidoreductase subunit H	2.1
BMMGA3_RS15425		NADH dehydrogenase subunit C	2.3
BMMGA3_RS15615	*mutB2*	methylmalonyl-CoA mutase	2.4
BMMGA3_RS15620		TetR/AcrR family transcriptional regulator	2.4
BMMGA3_RS15625	*acdA*	acyl-CoA dehydrogenase	2.2
BMMGA3_RS15630	*mmgC*	acyl-CoA dehydrogenase	2.6
BMMGA3_RS15635	*mmgB*	3-hydroxybutyryl-CoA dehydrogenase	2.6
BMMGA3_RS15640		acetyl-CoA C-acyltransferase	2.8

[a] Cut-off values set to a change in expression level higher than 30; $P \leq 0.01$, determined by Student's *t* test.
[b] Annotation according to Irla *et al*. (2016).
[c] Annotation according to this work's findings.
Differentially expressed genes belonging to the mannitol and arabitol operons are shown in bold.
BLASTx analysis results are shown in italics.

Supplementary Table 4. Growth rates, biomass yields and uptake rates of complemented *C. glutamicum* Δ*mtlD* strains grown on 30 mM arabitol.

**Strain and genotype**	**Growth rate (h^{-1})**	**Biomass yield (g CDW g^{-1})**	**Uptake rate (mmol g CDW^{-1} h^{-1})**
C. glutamicum Δ*mtlD*(pVWEx1-*atlABCD*)	0.10 ± 0.01	0.33 ± 0.02	1.9 ± 0.1
C. glutamicum Δ*mtlD*(pVWEx1-*atlD*)	0.11 ± 0.00	0.31 ± 0.01	2.3 ± 0.0
C. glutamicum Δ*mtlD*(pVWEx1-*atlABCDEF*)	0.11 ± 0.01	0.33 ± 0.03	2.2 ± 0.0

Supplementary Figures

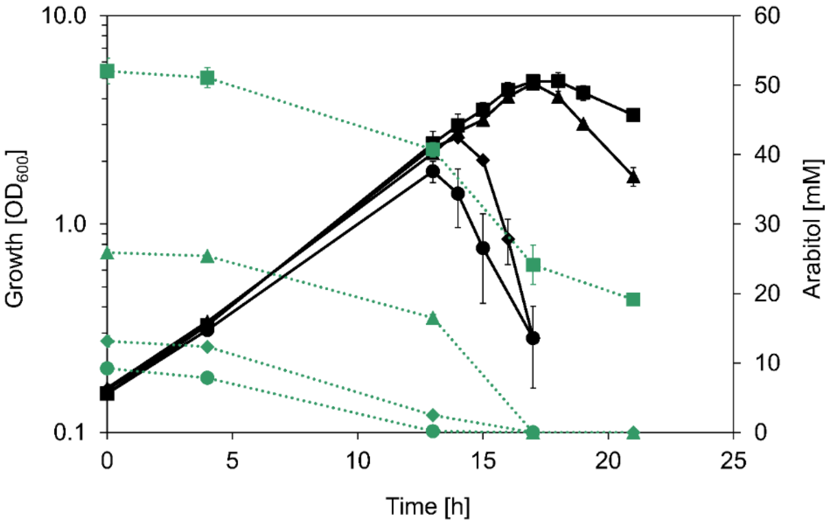

Supplementary Figure 1. Growth (black, solid lines) and substrate consumption (green, dotted lines) of *B. methanolicus* MGA3 in minimal media containing 10 mM (dots), 15 mM (diamonds) 30 mM (triangles) and 60 mM (squares) arabitol. Mean values and standard deviations of triplicate shake flask cultures are given.

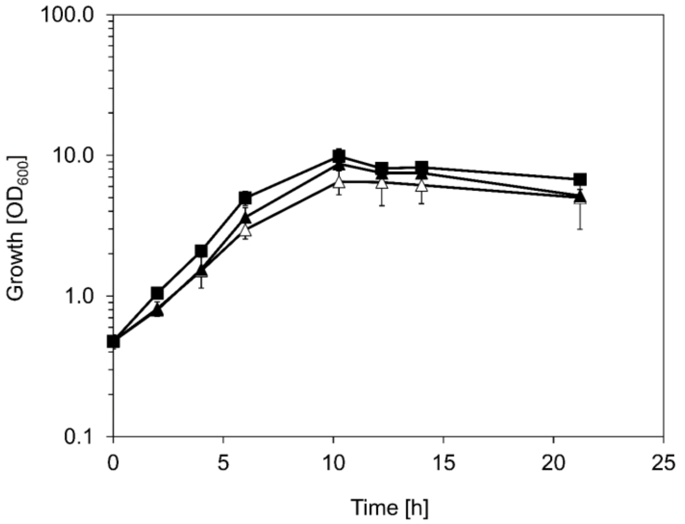

Supplementary Figure 2. Growth of *C. glutamicum* strains WT(pVWEx1) (full squares), Δ*mtlD*(pVWEx1) (empty triangles) and Δ*mtlD*(pVWEx1-*atlABCD*) expressing *atlABCD* genes from *B. methanolicus* MGA3 under IPTG induction (full triangles) in minimal media containing 30 mM glucose. Growth rates were determined to be 0.30 ± 0.01 h^{-1}, 0.26 ± 0.01 h^{-1} and 0.29 ± 0.00 h^{-1}, respectively. Mean values and standard deviations of triplicate shake flask cultures are given.

A

B

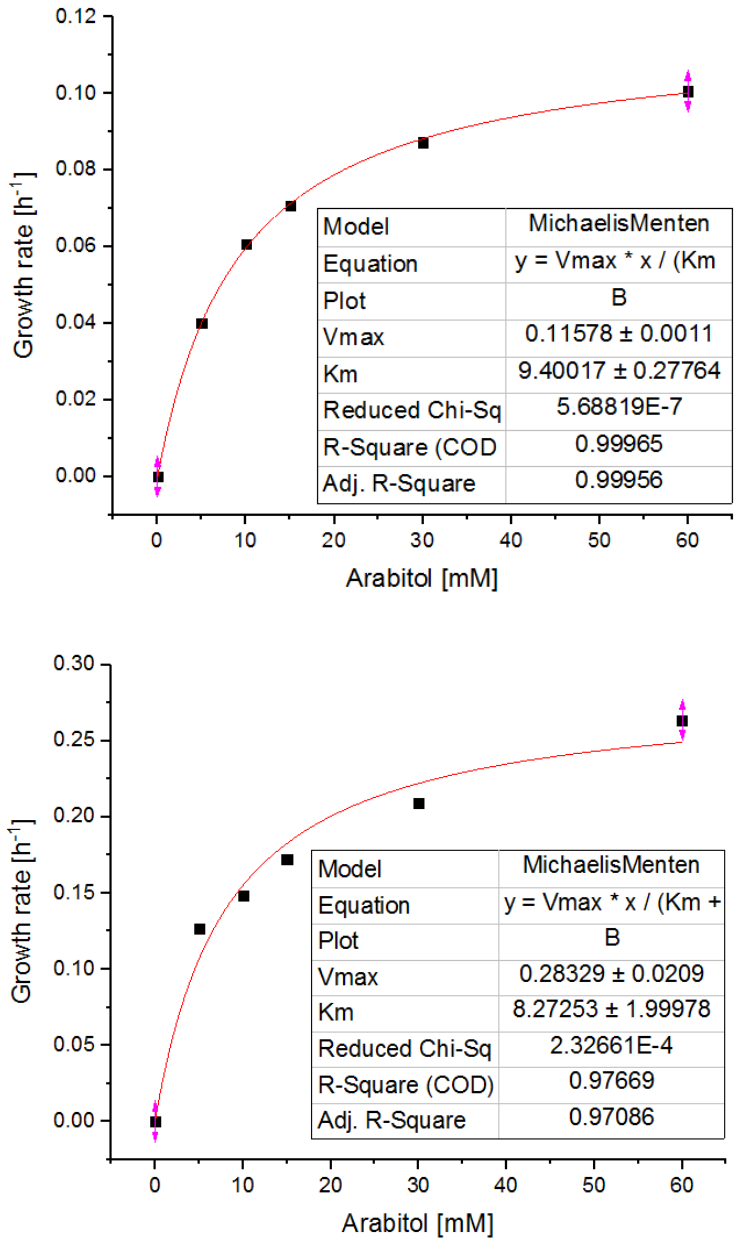

Supplementary Figure 3. Growth rates of *C. glutamicum* Δ*mtlD*(pVWEx1-*atlABCD*) **(A)** and *C. glutamicum* WT(pVWEx1) **(B)** in the presence of 5, 10, 15, 30 and 60 mM arabitol. A relation between growth rate and substrate concentration was generated with the Michaelis Menten model using the OriginPro software version 2018 (OriginLab Corporation, Northampton, MA, USA).

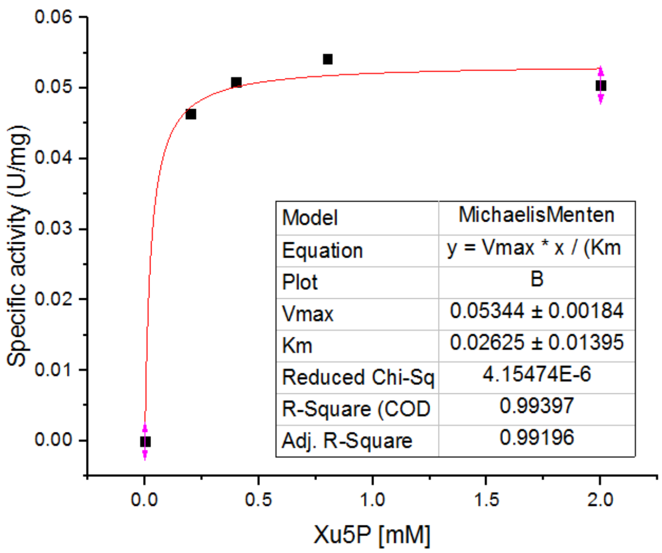

Supplementary Figure 4. Arabitol phosphate dehydrogenase activities of *B. methanolicus* crude extracts grown on arabitol in the presence of varying concentrations of xylulose 5-phosphte (Xu5P). Affinity for the substrate Xu5P was determined with the Michaelis Menten model using the OriginPro software version 2018 (OriginLab Corporation, Northampton, MA, USA).
